# Trojan horse in obstetric complications: A case report of concurrent uterine perforation, incarcerated fallopian tube and cesarean section scar pregnancy

**DOI:** 10.1097/MD.0000000000046539

**Published:** 2025-12-12

**Authors:** Ling Wang, XiangYu Chen

**Affiliations:** a Department of Ultrasound, Zhuji People’s Hospital of Zhejiang Province, Zhuji, China; b Department of Ultrasound, Zhuji Affiliated Hospital of Shaoxing University, Zhuji, China.

**Keywords:** cesarean scar pregnancy, incarcerated fallopian tube, uterine perforation

## Abstract

**Rationale:**

Uterine perforation complicated by incarcerated fallopian tube and cesarean scar pregnancy (CSP) constitutes an exceptionally rare and clinically challenging condition. The diagnostic dilemma arises from elevated β-hCG levels combined with parauterine mass formation, which frequently mimics tubal ectopic pregnancy presentation. This case report aims to highlight the critical importance of differential diagnosis in preventing clinical mismanagement of this complex obstetric emergency.

**Patient concerns:**

A 35-year-old woman (gravida 3, para 2) with recent Dilation and Curettage (D&C) presented with abdominal pain, vaginal bleeding, and elevated β-hCG, initially misdiagnosed as tubal ectopic pregnancy.

**Diagnoses:**

Initial transvaginal ultrasound detected a right adnexal mass with intrauterine fluid. Magnetic resonance imaging demonstrated communication between the mass and uterine myometrium. Repeat transvaginal ultrasound confirmed CSP, surgically validated.

**Interventions:**

Combined laparoscopic-hysteroscopic surgery performed uterine perforation repair, CSP resection, and right fallopian tube repositioning.

**Outcomes:**

The patient was discharged on postoperative day 7 without complications.

**Lessons:**

Heuristic-driven cognitive bias significantly contributes to diagnostic error. For multiparous women following abortion, heightened vigilance is essential to guard against the occurrence of this rare condition.

## 1. Introduction

Uterine perforation is a well-documented complication of intrauterine procedures including dilation and curettage (D&C) and hysteroscopy, with potential involvement of adjacent pelvic structures.^[1]^ Reported incidence varies by procedure type, ranging from 0.5% in first/second-trimester interventions to 5% in third-trimester procedures.^[2]^ Among the consequential anatomical disruptions, fallopian tube incarceration represents a clinically significant yet understudied phenomenon.^[3]^ Systematic reviews indicate that tubal incarceration may present across a broad temporal spectrum, from acute postoperative detection to delayed manifestations years after the initial event.^[4]^ This temporal variability complicates timely and accurate diagnosis of fallopian tubal incarceration.

This diagnostic complexity becomes particularly critical when fallopian tube incarceration coexists with cesarean scar pregnancy (CSP) – a high-risk ectopic implantation notorious for its propensity toward hemorrhagic complications and uterine rupture if undiagnosed.^[5]^ This anatomical complexity creates overlapping clinical presentations: both conditions may manifest as lateral uterine masses with elevated β-hCG levels, potentially leading to diagnostic inertia where this condition is misdiagnosed as tubal ectopic pregnancy.^[6]^ As conservative management is a viable option for tubal ectopic pregnancy, failure to detect concurrent CSP may precipitate life-threatening complications.^[7,8]^ To enhance practitioners’ awareness of this complex clinical entity, we present this case report.

## 2. Case presentation

A 35-year-old gravida 3 para 2 woman presented to the emergency department on Hospital Day 1 with acute-onset intermittent lower abdominal pain and persistent vaginal bleeding lasting 24 hours. Three months earlier, she underwent D&C for a nonviable intrauterine pregnancy at an estimated 8 weeks gestation based on ultrasound. A follow-up transvaginal ultrasound (TVUS) 1 week post-procedure revealed an approximately 3.62 × 1.57 cm heterogeneous echogenic fluid collection within the uterine cavity, interpreted as retained products of conception or hematoma, managed expectantly. The patient was subsequently managed with medical therapy with close monitoring.

Her gynecological history was negative for pelvic inflammatory disease, sexually transmitted infections, or endometriosis but included 2 prior lower-segment cesarean deliveries. She reported no fertility evaluations but expressed concerns about irregular cycles post-surgery. The patient could not recall her last menstrual period. Her medical history was unremarkable, with no chronic diseases, regular medications, allergies, or tobacco use.

**Figure 1.**
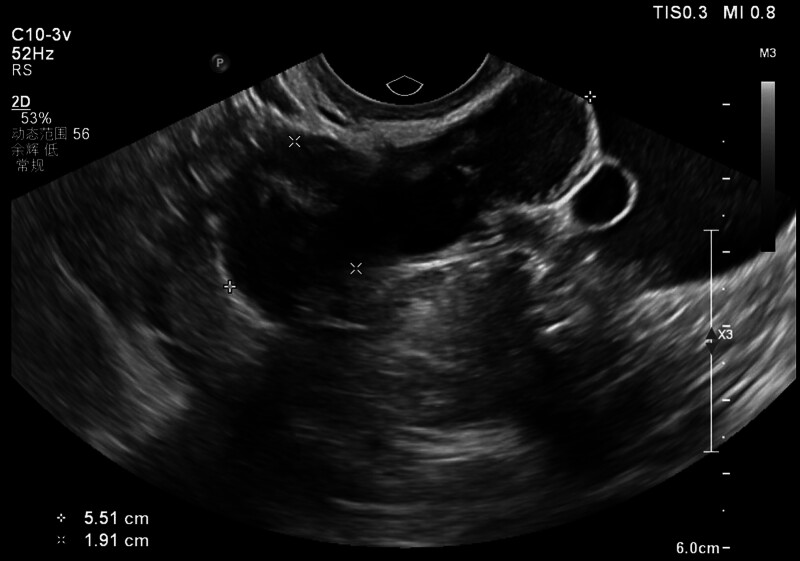
Transvaginal ultrasonography demonstrated a heterogeneous hyperechoic adnexal mass (5.51 × 1.91 cm) in the right pelvic region.

**Figure 2.**
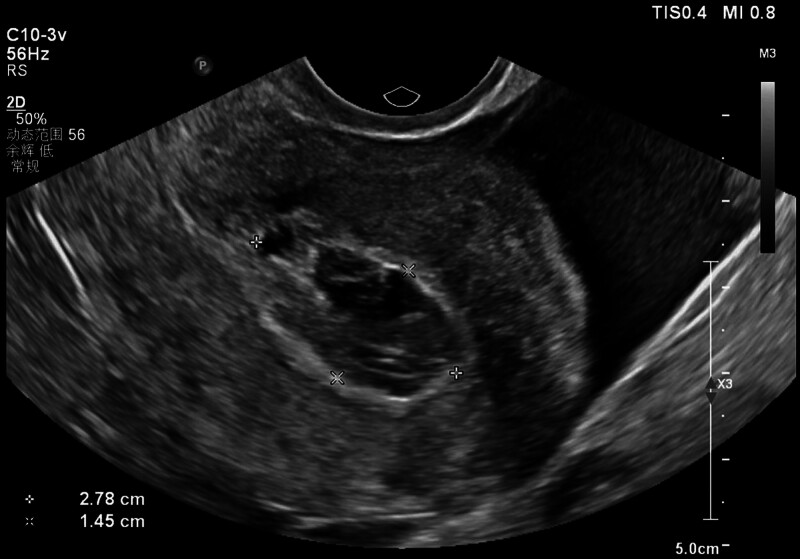
Transvaginal ultrasonography revealed a heterogeneous hyperechoic intrauterine fluid collection (2.78 × 1.45 cm).

On hospital day 1, physical examination demonstrated stable vital signs and a soft abdomen with mild right lower quadrant tenderness. The cervical os was closed without motion tenderness. The initial serum β-hCG level was 372 IU/L. The discrepancy between uterine size and β-hCG level, combined with her history, raised suspicion for ectopic pregnancy or pregnancy of unknown location. TVUS revealed a complex 5.51 × 1.91 cm right adnexal mass with cystic and solid components (Fig. 1), alongside a 2.78 × 1.45 cm echogenic heterogeneous uterine cavity fluid collection (Fig. 2). The TVUS report indicated right tubal ectopic pregnancy and possible intrauterine hematocele.

**Figure 3.**
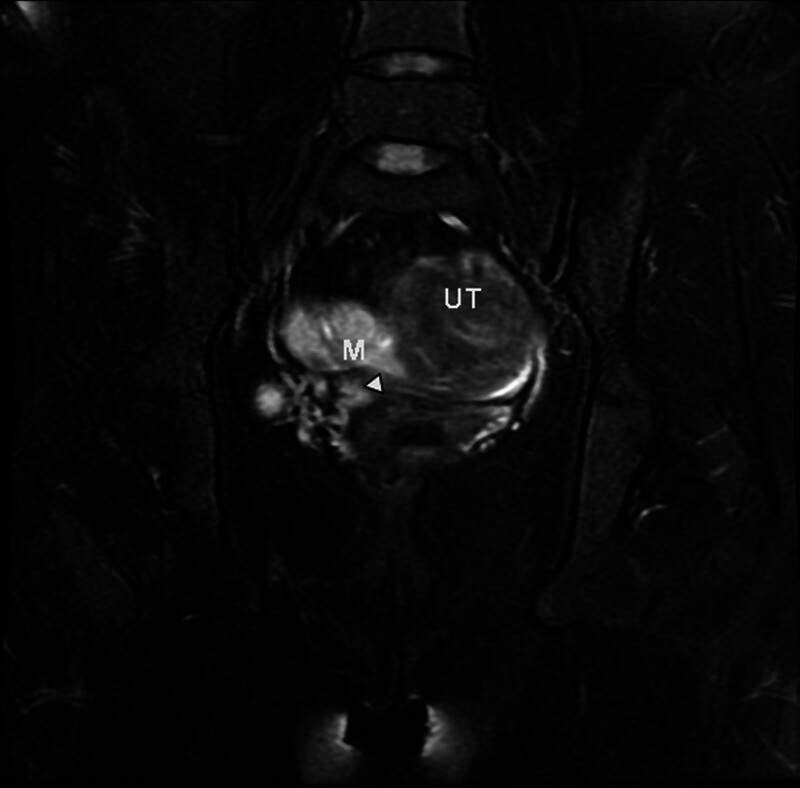
Magnetic resonance imaging (MRI) coronal section demonstrated a right parametrial mass of adnexal origin with myometrial invasion (arrow indicates herniated segment of the ipsilateral fallopian tube within the uterine musculature). UT = uterus, M = mass.

**Figure 4.**
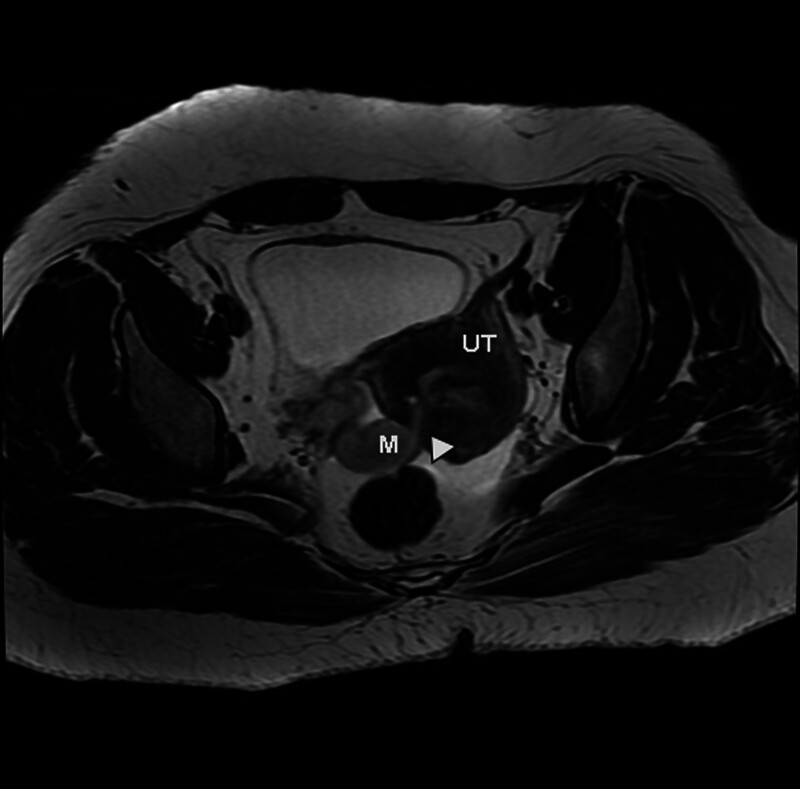
Magnetic resonance imaging (MRI) on axial section exhibited a right parametrial mass of adnexal origin with myometrial invasion (arrow indicates herniated segment of the ipsilateral fallopian tube within the uterine musculature). UT = uterus, M = mass.

**Figure 5.**
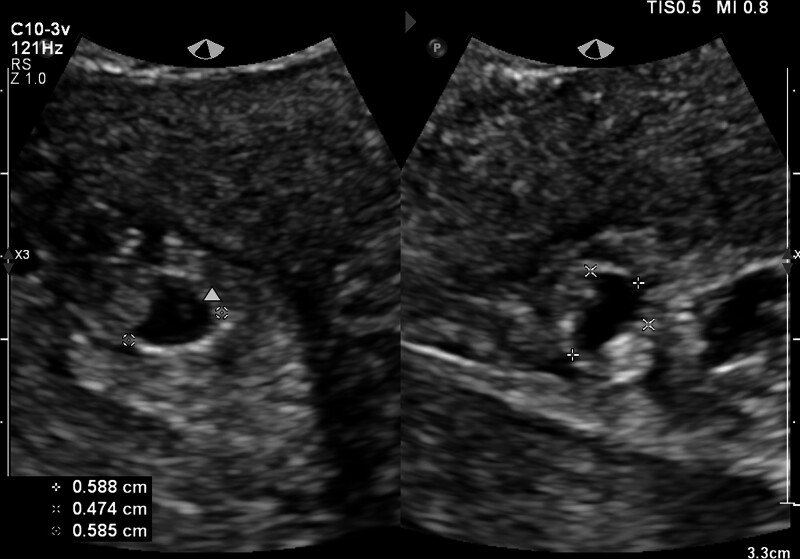
Transvaginal ultrasonography reexamination revealed a gestational sac echo near the cesarean section incision in the lower uterine segment, about 0.588 × 0.474 × 0.585 cm (*) in size, with a yolk sac inside. Arrow: yolk sac.

**Figure 6.**
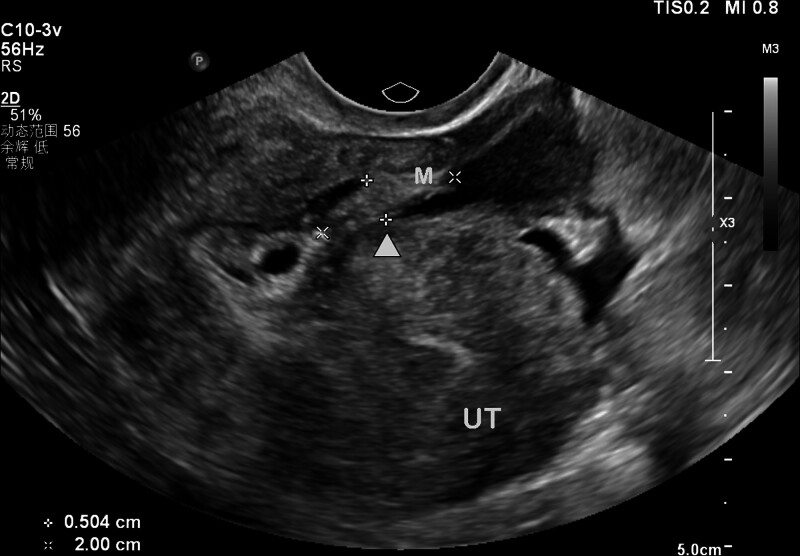
Transvaginal ultrasound demonstrates a 2.00 × 0.50 cm hypoechoic mass (*) originating from the right parauterine region, with intramyometrial extension. Arrow indicates the interface between parauterine and myometrial components. UT = uterus, M = mass.

On Hospital Day 2, repeat β-hCG had risen minimally to 415 IU/L over 24 hours – an increase of only 11.6%, which falls below the threshold expected for a viable intrauterine pregnancy (typically > 53–66% over 48 hours) and further undermined the likelihood of normal gestational development.^[8]^ Despite the suboptimal β-hCG rise and a sizable adnexal mass measuring up to 5 cm, the patient remained hemodynamically stable, which was inconsistent with the typical clinical presentation of ruptured tubal ectopic pregnancy. This discrepancy raised the possibility of alternative pathologies. Pelvic magnetic resonance imaging (MRI) was subsequently arranged to further evaluate the condition. Pelvic MRI confirmed a right adnexal mass extending into and invading the myometrium (Figs. 3 and 4). Given the history of CSP, the adnexal mass may represent an incarcerated fallopian tube; the CSP might be missed. Subsequent targeted TVUS identified a gestational sac with yolk sac embedded within the previous cesarean incision site at the isthmic portion of the anterior lower uterine segment (Fig. 5). A 2.0 × 0.5 cm strip-like heterogeneously hypoechoic focus was identified embedded within the right uterine myometrium, demonstrating an echo texture resembling that of the fallopian tube (Fig. 6). Notably, upon retrospective review of the initial TVUS images, the sonographer observed that the right adnexal mass exhibited well-demarcated borders and predominantly cystic fluid-filled internal echogenicity, which differed from the typical features of a ruptured tubal ectopic pregnancy.

The patient underwent emergency combined laparoscopic and hysteroscopic surgery due to the simultaneous presence of a parametrial mass and CSP. Laparoscopy identified a 1 cm defect in the right uterine segment with partial fallopian tube incarceration (approximately 2.5 cm in length). Hysteroscopy confirmed the CSP implantation. The CSP lesion was resected, followed by anatomical repositioning of the incarcerated fallopian tube, and the uterine defect was repaired. Hemostasis was achieved with bipolar cautery and suture ligation. Estimated blood loss was 300 mL; no transfusion required. Histopathology confirmed gestational tissue. Postoperative TVUS demonstrated no parametrial mass, intrauterine fluid collection, or residual CSP tissue.

Her postoperative recovery was uncomplicated. Serial β-hCG levels declined to 92 IU/L on day 1, 35 IU/L on day 3, and were undetectable at 2 weeks. She was discharged on postoperative day 7. At 6-week follow-up, symptoms had fully resolved, with normal menstruation resumed by 5 weeks. A 3-month TVUS showed no identifiable mass or residual tissue at the uterine incision site, with no sonographic evidence of CSP. Subsequent fertility assessment revealed no impairment of reproductive function.

## 3. Discussion

D&C remains essential in gynecological practice for therapeutic abortion, postoperative hemostasis, specimen collection, and uterine evacuation. Despite proven efficacy, all intrauterine procedures carry uterine perforation risks, remaining a safety concern regardless of complexity.^[9]^ A retrospective analysis revealed uterine perforation rates of 0.38% (15/3913) in D&C procedures.^[10]^ Uterine perforations following D&C procedures may disrupt pelvic anatomy through structural displacement or tissue incarceration. Intrauterine fallopian tube incarceration, a rare complication, lacks pathognomonic symptoms. Diagnosis is challenging due to nonspecific clinical indicators, requiring imaging confirmation in suspected cases.^[4]^

The prevalence of CSP correlates with rising global cesarean section rates, now reaching 21.1% worldwide.^[11]^ While epidemiological studies report a CSP incidence of 1 per 1688 pregnancies, accumulating clinical evidence highlights substantial underdiagnosis attributed to inherent limitations in routine obstetric detection modalities.^[12]^ Notably, CSP detected before 10 weeks of gestation is often incidentally identified during routine ultrasound screenings in asymptomatic women.^[13]^

When serum β-hCG levels < 1500 mIU/mL and no intrauterine gestational sac is identified on TVUS, ectopic pregnancy should be strongly suspected.^[14]^ CSP is a special type of ectopic pregnancy, though current guidelines lack definitive β-hCG threshold ranges for its diagnosis, and it is generally managed within the framework of ectopic pregnancy protocols.^[8,15]^ If CSP is suspected, prompt diagnosis with TVUS is recommended, ideally around 6 to 7 weeks of gestation.^[16]^

Tubal incarceration secondary to uterine perforation and early-stage CSP present with indistinguishable clinical features in emergency settings, posing significant diagnostic challenges in contemporary obstetric practice.^[17]^ The combination of the patient’s 2 previous cesarean deliveries and recent post-miscarriage D&C procedure constituted significant predisposing factors for uterine perforation and CSP.

CSP is classified into 3 distinct subtypes based on the spatial relationship between the gestational sac and key uterine anatomical landmarks^[18]^ (Table 1). Although this CSP case was categorized as the most common subtype (endocavitary), concurrent fallopian tube incarceration contributed to diagnostic uncertainty. The diagnosis was initially confounded by heterogeneous echogenic intrauterine fluid obscuring sac topography. Concurrent findings of a adnexal mass with heterogeneous internal echogenicity and elevated β-hCG levels further reinforced the initial misdiagnosis of tubal ectopic pregnancy, as the sonographic features closely mimicked those of a ruptured tubal hematoma. Additionally, the patient’s recent history of abortion made it impossible to rule out the presence of intrauterine pregnancy residues, further complicating the diagnosis. The differential diagnosis also included hCG-secreting adnexal tumors, which often lack pathognomonic imaging characteristics. Retrospective imaging analysis revealed the extrauterine mass lacked typical ectopic pregnancy features, whereas on targeted TVUS, a hypoechoic structure compatible with gestational sac morphology was identified in the lower uterine cavity near the isthmus. A CSP concealed within intrauterine fluid—akin to a “Trojan horse” of obstetric peril—poses a dual threat: its latent progression risks catastrophic uterine rupture, while preoperative misdiagnosis as tubal ectopic pregnancy may trigger unnecessary surgical cascades, compounding patient morbidity.

**Table 1:** Classification of cesarean scar pregnancy (CSP) subtypes.

Subtype	Key characteristics
Endocavitary	Most common type; >50% of gestational sac projects into the uterine cavity.
Intramural	Gestational sac completely confined within the myometrium, without endometrial or serosal invasion.
Exophytic	Gestational sac extends beyond the uterine serosa, often protruding into the bladder wall.

The most dangerous complication of CSP is uterine rupture, which can manifest as acute severe pain, massive bleeding, and hemoperitoneum, leading to hemodynamic shock.^[19]^ Taking into account that the patient suffered from CSP, along with uterine perforation, as well as incarceration of surrounding organs – which could potentially lead to severe hemodynamic-affecting complications – a combined hysteroscopic-laparoscopic surgical approach was implemented. Given the suspected presence of micro-uterine rupture, neither methotrexate therapy nor uterine artery embolization would have been appropriate, as these conservative measures risk exacerbating occult perforation or hemorrhage.^[20]^

With timely diagnosis and treatment, both uterine perforation and CSP generally have a favorable prognosis.^[21]^ Upon completion of desired childbearing, comprehensive contraceptive counseling should be provided, with particular emphasis on long-acting reversible contraceptives. This discussion must be evidence-based and patient-centered, incorporating individual risk factors for recurrent abnormal pregnancies.^[22]^

For comprehensive postoperative fertility surveillance, a multimodal imaging approach is recommended, including: serial transvaginal sonography at 1, 3, and 6 months to evaluate cesarean scar healing dynamics; saline infusion sonohysterography or hysteroscopy for detailed endometrial cavity assessment when indicated; selective MRI for suspected deep myometrial defects; and Hysterosalpingography may be reserved for patients with future fertility plans to concurrently assess tubal patency.^[23]^

## 4. Conclusion

Uterine perforation with incarcerated fallopian tube and concurrent CSP constitutes a clinically deceptive entity – reminiscent of a “Trojan horse” – that harbors life-threatening risks requiring prompt multidisciplinary management. Clinicians must challenge diagnostic biases to enhance early recognition. Radiologists and sonographers should carefully assess ultrasound findings, particularly the anatomical continuity between adnexal masses and the uterine cavity. In women with prior cesarean delivery and elevated β-hCG, CSP must be definitively ruled out irrespective of symptomatology.^[24]^

## Author contributions

**Conceptualization:** XiangYu Chen.

**Writing – original draft:** Ling Wang.

**Writing – review & editing:** XiangYu Chen.
